# *Circumfusicillium cavernae* gen. et sp. nov. (*Bionectriaceae*, *Hypocreales*) Isolated from a Hypogean Roman Cryptoporticus

**DOI:** 10.3390/jof8080837

**Published:** 2022-08-10

**Authors:** João Trovão, Fabiana Soares, Diana Sofia Paiva, Igor Tiago, António Portugal

**Affiliations:** 1 Centre for Functional Ecology, Department of Life Sciences, University of Coimbra, Calçada Martim de Freitas, 3000-456 Coimbra, Portugal; 2 Fitolab-Laboratory for Phytopathology, Instituto Pedro Nunes, 3030-199 Coimbra, Portugal

**Keywords:** biodeterioration, fungi, hypogean environments, limestone, new taxa, systematics

## Abstract

Stone monuments and relics are prone to biodeterioration processes prompted by microbial proliferation and activity. Among the distinct microbes capable of stone colonization, fungi are known to strongly contribute to stone biodeterioration. During the ongoing efforts aiming to study fungi thriving in dolomitic limestone walls of the Coimbra’s hypogean Roman cryptoporticus (Portugal), two unknown *Bionectriaceae* isolates were retrieved. The aim of this work was to depict the molecular and phenotypic characteristics of these microorganisms. The phylogenetic analyses revealed that the studied strains could not be assigned to any of the currently known *Bionectriaceae* genera. Moreover, the isolates exhibited distinctive and peculiar characteristics, such as the packing of conidia by surrounding hyphal segments and the formation of rope-like microsclerotia with a *textura globose*. Taking into account all the data obtained, a novel genus and species, *Circumfusicillium cavernae* gen. et sp. nov. in *Bionectriaceae* (*Hypocreales*), is proposed here.

## 1. Introduction

Hypogean cultural heritage monuments are often exposed to extreme conditions [1,2,3,4], with the fungal community under these circumstances being strongly shaped by other microbes (e.g., photoautotrophs) and external factors (e.g., animal vectored dispersion, water availability and anthropogenic activities) [4,5,6]. When dealing with stone monuments, fungi are known to be one of the most important biodeteriorative microorganisms since they can deeply alter stone integrity, structural and aesthetic characteristics [7,8,9]. Fungi contribute to stone biodeterioration through various physical and chemical mechanisms that can result in pitting, mineral dislocation, dissolution and reprecipitation [8,9,10,11,12]. Such processes can unwittingly cause irreversible cultural heritage losses. However, the fungal diversity in hypogean environments and monuments has also been pinpointed as largely unexplored, a situation that hampers the proper application of suitable conservation treatments aiming to preserve such cultural heritage materials for future generations since these microorganisms’ biocide susceptibilities remain uncharacterized [13].

The *Hypocreales* is a largely diversified fungal order, currently comprising fifteen accepted families, namely *Bionectriaceae*, *Calcarisporiaceae*, *Clavicipitaceae*, *Cocoonihabitaceae*, *Cordycipitaceae*, *Cylindriaceae*, *Flammocladiellaceae*, *Hypocreaceae*, *Myrotheciomycetaceae*, *Nectriaceae*, *Niessliaceae*, *Ophiocordycipitaceae*, *Sarocladiaceae*, *Stachybotryaceae* and *Tilachlidiaceae* [14,15]. Species in this order exhibit highly versatile strategies to exploit their substrata and are often able to survive under various differential environments [16,17]. *Bionectriaceae* currently encompasses one of the largest fungal families in the order *Hypocreales*, classically being considered to include genera that have uniloculate, perithecial, rarely cleistothecial ascomata that are white, orange or brown, not changing color in KOH [17]. Some species with vastly diverse ecologies and exhibiting acremonium-like or verticillium-like anamorphic characters are also present in this family [18,19].

With the ongoing characterization of fungal limestone biodeterioration phenomena in the UNESCO World Heritage site of “University of Coimbra—Alta and Sofia” (Coimbra, Portugal), various studies are also being conducted in the city’s Roman hypogean cryptoporticus (first or second century AD) (e.g., [20]). One survey aiming to isolate fungal species thriving in this monument allowed the retrieval of two unknown *Bionectriaceae* strains from a mature biofilm. Therefore, the aim of this work was to determine the taxonomic position of this fungus by employing phylogenetic analyses coupled with morphological examinations. This integrative analysis highlighted that this fungus represents a novel genus and species in *Bionectriaceae*, here proposed and described as *Circumfusicillium cavernae* gen. et sp. nov.

## 2. Materials and Methods

### 2.1. Site Description and Fungal Isolation

The Machado de Castro National Museum (Museu Nacional Machado de Castro—MNMC) is one of the most important Portuguese art repositories, holding a vast asset of ancient national religious artworks. The museum is located in the former city Bishop’s palace, built during the Middle Age. The site corresponds to where the Roman forum of Aeminium (Coimbra’s ancient name) once stood. The cryptoporticus is located underneath the museum, being composed of an underground gallery of arched dolomitic limestone corridors built in the first or second century AD. Gaius Sevius Lupus is thought to be the architect responsible for its construction. In the south of the cryptoporticus, a large sewer flowing to the Mondego River is present, likely representing the former *cloaca maxima* of Aeminium.

Sampling was conducted in the cryptoporticus cells (large communicating areas leading to a gallery), with the samples being obtained as described previously [20,21], by scraping small areas from a well-developed biofilm in a deteriorated limestone wall with a sterile scalpel into a collection tube. All sampling procedures were performed with the permission of “Direcção Regional de Cultura do Centro” (DRCC, local Government authority) and supervised by technicians from the MNMC. This sampling site was characterized by having a temperature of 23 °C ± 1, humidity of 56%, light intensity of 66 lx and by visible water dripping down the walls [20]. Complementarily, we have previously found that this area is largely dominated by the phototrophs *Asterionella*, *Geitlerinema*, *Mastigocladopsis*, Oscillatoriales and *Pleurocapsa*, and by fungi belonging to *Cephalotrichum*, *Chaetothyriales*, *Cyphellophora*, *Lecanicillium* and *Mortierella* [20].

Sample inoculation was performed after the suspension of the retrieved rocky material in 3 mL of sterile 0.9% (*w*/*v*) NaCl solution, vortexing and plating over Potato Dextrose Agar (PDA) (Difco, New Jersey, USA) supplemented with streptomycin (0.5 g L^−1^) [20]. Plate incubation was performed for a period of thirty days at room temperature (27 ± 1 °C) and in the dark [20]. The emerging *Bionectriaceae* colonies were isolated to similar fresh media and further incubated until biomass had developed for DNA extraction (circa fifteen days).

### 2.2. DNA Extraction, PCR Amplification, Sequencing and Phylogenetic Analyses

Total genomic DNA extraction of the two *Bionectriaceae* isolates was conducted with the REDExtract-N-Amp Plant PCR Kit (Sigma-Aldrich, St. Louis, USA) as previously described [20,21]. The genomic DNA obtained was subjected to PCR amplification of the Internal Transcribed Spacer region (ITS) and the 28S gene (LSU), with a program composed of 35 cycles, with an initial denaturation temperature of 94 °C for 1 min, primer annealing at 55 °C for 1 min, extension at 72 °C for 1 min, and a final extension step at 72 °C for 5 min. PCR mixes contained a final volume of 25 µL, with 12.5 µL of NZYTaq Green Master Mix (NZYTech™, Lisboa, Portugal), 1 µL of each primer (10 mM), 9.5 µL of ultra-pure water and 1 µL of template DNA. For the amplification of the ITS rDNA region, the primer pair ITS1-F/ITS4 [22,23] was used, while the amplification of the LSU region was achieved with the primer pair LSU1fd/LR5R [24,25]. All PCR reactions were conducted in an ABI GeneAmp™ 9700 PCR System (Applied Biosystems, Waltham, USA). Purification of the amplified amplicons was conducted with the NZYGelpure DNA purification kit (NZYTech™, Lisboa, Portugal) and then sequenced using an ABI 3730xl DNA Analyzer system (96 capillary instruments) at STABVIDA, Portugal.

DNA sequences were quality checked and assembled using the Geneious^®^ R11.0.02 software (https://www.geneious.com) and deposited in GenBank (see Table 1). The obtained sequences were initially compared with the sequences available in the National Center of Biotechnology Information nucleotide database using NCBI’s Basic Local Alignment Search Tool (BLAST), with the option standard nucleotide Blastn [26]. To further evaluate the *Bionectriaceae* isolate’s phylogenetic position, three datasets consisting of partial LSU (dataset 1), partial ITS (dataset 2) and concatenated ITS and LSU (dataset 3) reference sequences were constructed (adapted from [27,28], see Table 1). In addition, considering that some GenBank “uncultured” sequences and isolates identified as *Geosmithia* sp. (to the best of our knowledge, not formally described) had a significant ITS Blast result (>95%), a smaller dataset (dataset 4) was also constructed to include and study these sequences. For each dataset, sequences were individually aligned using the online version of MAFFT v.7 [29] and manually adjusted using UGENE v.1.26.3 [30]. For the construction of dataset 3, the individual alignments were concatenated with SeaView v.4 [31]. Prior to the phylogenetic analysis, the model of nucleotide substitution was estimated under the Akaike Information Criterion (AIC) using MrModeltest v.2.3 [32] (for all cases nst = 6 rates = invgamma). Phylogenetic analysis was conducted considering both Maximum likelihood and Bayesian methods. The Maximum likelihood analysis was conducted using RaxmlGUI v.2.0.0 with 1000 bootstrap replicates [33]. In parallel, a Bayesian MCMC analysis was performed using MrBayes v.3.2.6 [34] for four runs, ten million generations, heated chain “temperature” of 0.15 and trees being saved after every 100 generations. Upon the analysis conclusion, Tracer v.1.5 [35] was used to ensure that convergence had been reached. The burn-in phase (25%) was discharged, and the remaining trees were used to calculate the Bayesian posterior probabilities (BP) in a 50% majority rule consensus tree that was then viewed in FigTree v.1.2.2 [36]. For dataset 1, the tree was rooted with *Neocosmospora vasinfecta* (GenBank accession number U17406); for datasets 2 and 4, the trees were rooted with *Verticillium bulbillosum* (GenBank accession number NR_154142), while for dataset 3, the tree was rooted with *Thyronectria rhodochlora* (GenBank accession numbers MH877605 and KJ570704). All the obtained alignments and phylogenetic trees were deposited in figshare (10.6084/m9.figshare.20279658).

### 2.3. Morphological Analysis

The isolates were grown in unfiltered Oatmeal Agar (OA) (60 g of oatmeal flakes, 12.5 g of agar, 1 L water) for twenty-one days and microscopical analysis was performed directly or using the slide culture technique, with the slides being stained with lactophenol cotton blue (Sigma-Aldrich, St. Louis, USA). Microscopical observations were conducted with a light microscope and photographed (Leica DM 4000B + Leica DFC 490 digital camera (Leica, Wetzlar, Germany)). A holotype and ex-type living cultures were deposited in Micoteca da Universidade do Minho (MUM), Braga, Portugal.

## 3. Results

### 3.1. Phylogenetic Analyses

Initial comparisons with the sequences deposited in the NCBI database revealed that the similarity with the closest reference organisms for the ITS sequences was 92% (*Stilbocrea macrostoma* CLLG18056), while for the LSU sequences was 98% (*Geosmithia xerotolerans* FMR 17085), showing that the isolates belonged to the family *Bionectriaceae.*

**Figure 1:**
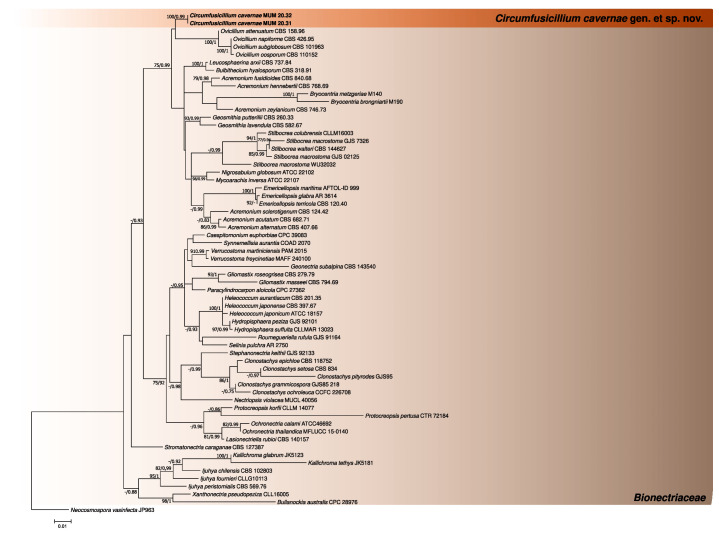
Phylogenetic trees obtained using reference *Bionectriaceae* LSU sequences. The new taxa are indicated in **black** and **bold**. The scale bar indicates the number of substitutions per site, and the support values (>75% bootstrap values for Maximum likelihood and >0.75 for Bayesian MCMC posterior probabilities) are also shown.

**Figure 2:**
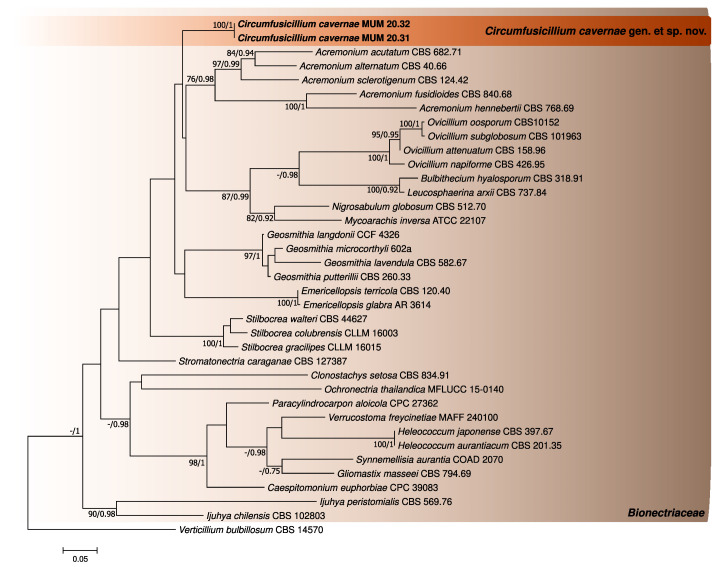
Phylogenetic trees obtained using reference *Bionectriaceae* ITS sequences. The new taxa are indicated in **black** and **bold**. The scale bar indicates the number of substitutions per site, and the support values (>75% bootstrap values for Maximum likelihood and >0.75 for Bayesian MCMC posterior probabilities) are also shown.

**Figure 3:**
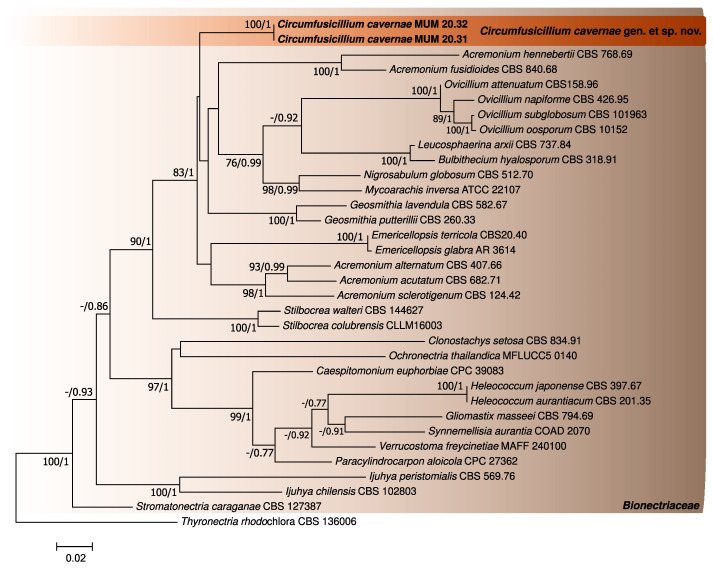
Phylogenetic trees obtained using the concatenated ITS and LSU reference *Bionectriaceae* sequences. The new taxa are indicated in **black** and **bold**. The scale bar indicates the number of substitutions per site, and the support values (>75% bootstrap values for Maximum likelihood and >0.75 for Bayesian MCMC posterior probabilities) are also shown.

The phylogenetic analyses performed for the three first datasets were constructed using individual partial LSU and ITS sequence alignments and a concatenated matrix of *Bionectriaceae* reference sequences (802 (LSU), 540 (ITS) and 1317 (concatenated matrix) nucleotides, including alignment gaps, respectively). For each case, the generated trees from Bayesian and Maximum likelihood analyses showed similar topologies between them, being in accordance with the current knowledge regarding this family [27] (Figure 1, Figure 2 and Figure 3). In addition, the phylogenetic analyses revealed that the studied fungus could not be properly assigned to any of the currently known *Bionectriaceae* genus, forming a separate lineage closely affiliated with *Ovicillium* (introduced by Zare and Gams [18]), according to the LSU analysis.

**Figure 4:**
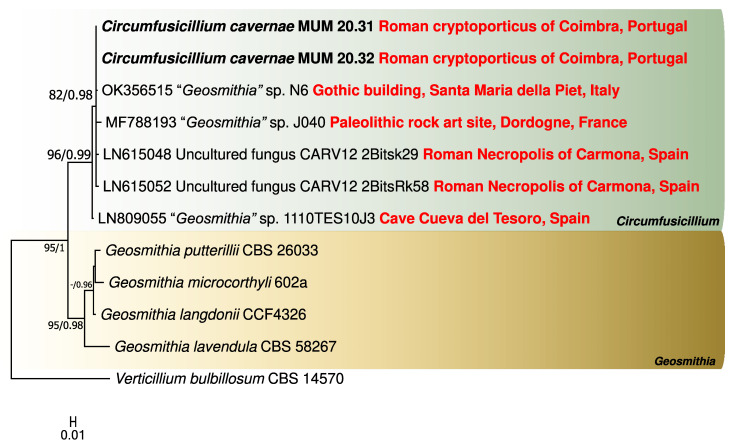
Phylogenetic trees obtained using best Blast hits ITS sequences and *Geosmithia* representatives. The new taxa are indicated in **black** and **bold**. The original study sites are presented in **bold and red**. The scale bar indicates the number of substitutions per site, and the support values (>75% bootstrap values for Maximum likelihood and >0.75 for Bayesian MCMC posterior probabilities) are also shown.

The phylogenetic analyses on dataset 4 also allowed the verification that the GenBank ITS sequences labeled as “uncultured” and as *Geosmithia* sp. clustered with the sequences generated in this study. Since these microorganisms/sequences have been found associated with cave and other monument biodeterioration scenarios [37,38,39,40], these results further highlight *Circumfusicillium* ecology and putative geographical distribution (Figure 4).

### 3.2. Morphological Analysis

**Taxonomy**

*Bionectriaceae* Samuels and Rossman

**Figure 5:**
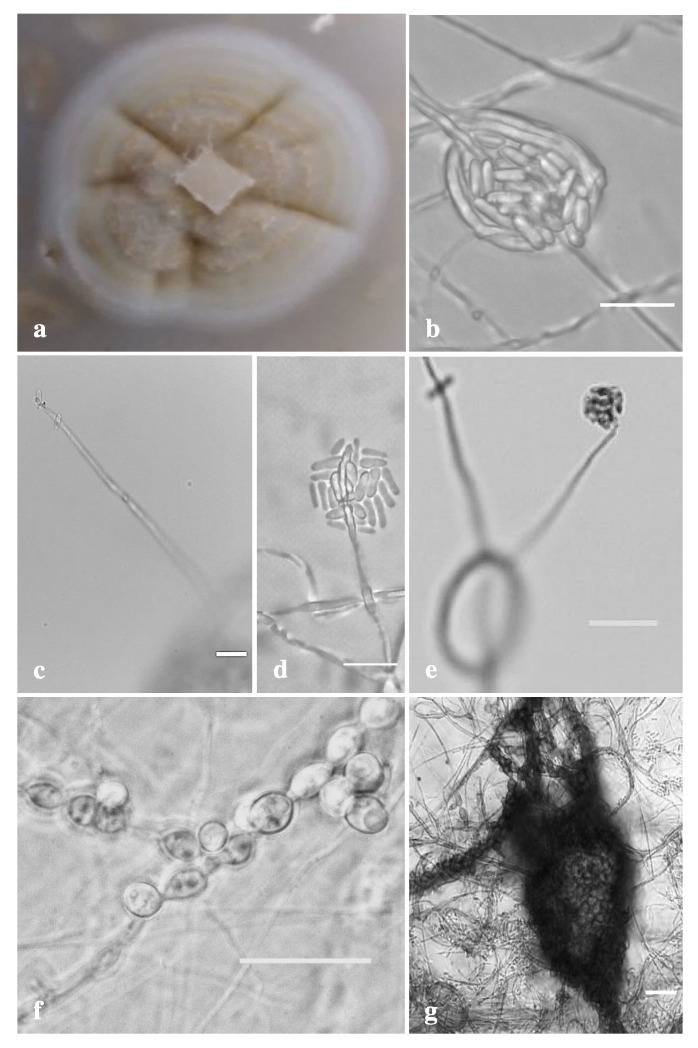
*Circumfusicillium cavernae* details of the: (**a**) Colony characteristics on OA after 21 days; (**b**) Conidia tightly packed by surrounding hyphal segments; (**c**–**e**) Typical conidiophores, conidial heads and conidia; (**f**) Typical chlamydospore chains; and (**g**) Chains of chlamydospores interweaving in long rope-like microsclerotia with *textura globose*. Scale bars (**b**–**e**) 10 μm; (**f**,**g**) 20 μm.

***Circumfusicillium*** J. Trovão, F. Soares, D.S. Paiva and A. Portugal, **gen. nov.** (Figure 5).

MycoBank number: MB834762.

*Etymology*: From “circumfusus” denoting the sometimes-visualized packing of conidia by surrounding hyphal segments.

*Type species*: *Circumfusicillium cavernae* J. Trovão, F. Soares, D.S. Paiva & A. Portugal.

*Description*: Asexual morph, hyphae hyaline to subhyaline, smooth, thin-walled, solitary or forming hyphal ropes and coils. Simple phialidic conidiophores, hardly distinguishable from hyphae, smooth, tapering to the tip. Conidia arranged mostly in terminal heads. Solitary conidia can also be formed at hyphal tips. In hyphal coils, conidia sometimes can become tightly packed by surrounding hyphal segments. Conidia unicellular, hyaline to subhyaline, smooth, narrowly cylindrical. Chlamydospores mostly developing in intercalary chains, initially hyaline to subhyaline turning brown to dark brown later, globose to subglobose, cyanophilic, smooth, thick-walled. Chlamydospore chains interweaving in long rope-like microsclerotia, with cells of microsclerotia globose to subglobose, forming a *textura globose*. Sexual morph unknown.

***Circumfusicillium cavernae*** J. Trovão, F. Soares, D.S. Paiva and A. Portugal, **sp. nov.** (Figure 5).

MycoBank number: MB834763.

*Etymology*: Denoting the “show-cave”-like environment where the isolates were retrieved.

*Typification*: Portugal, Coimbra (40°12′31.69″ N, 8°25′31.63″ W) isolated from a biofilm covering a biodeteriorated limestone wall in the Machado de Castro National Museum Cryptoporticus, 4 April 2019, J. Trovão, (holotype MUM-H 20.31, dried specimen), ex-type culture MUM 20.31.

*Description*: Hyphae hyaline to subhyaline, 1–3 μm wide, smooth, thin-walled, solitary or forming hyphal ropes and coils. Asexual morph, simple phialidic conidiophores, hardly distinguishable from hyphae, cyanophilic, smooth, tapering to the tip, reaching up to 100 μm long. Conidia arranged mostly in terminal heads, 6.5–14.5 × 6.5–19 μm wide. Often, solitary conidia are also formed at hyphal tips. Moreover, in hyphal coils, conidia sometimes can become tightly packed by surrounding hyphal segments. Conidia unicelular, hyaline to subhyaline, cyanophilic, smooth, narrowly cylindrical (rod-shaped), 3–4.8 × 1.3–2.6 μm wide. Chlamydospores mostly developing in intercalary chains, initially hyaline to subhyaline turning brown to dark brown later, globose to subglobose, cyanophilic, smooth, thick-walled, 3–8 × 3–6.5 μm wide. Chlamydospore chains interweaving in long rope-like microsclerotia, highly variable in size and often occupying entire portions of the colony. Cells of microsclerotia globose to subglobose, forming a *textura globose*. Sexual morph unknown.

*Colony characteristics*: After 21 days at 25 °C on OA, colonies growing slowly, reaching up to 15 mm in diameter, slightly raised at center, crateriforme with radial waves, scarce whitish to greyish aerial mycelium, margins entire, narrow (1–2 mm), sporulation abundant, chlamydospores present often immersed into the agar. Colonies white to yellow-brown on top and reverse. Crystals, exudates and diffusible pigment absent.

*Additional specimens examined*: Portugal, Coimbra (40°12′31.69″ N, 8°25′31.63″ W) isolated from a biofilm covering a biodeteriorated limestone wall in the Machado de Castro National Museum Cryptoporticus, 4 April 2019, J. Trovão, MUM 20.32.

*Notes*: *Circumfusicillium cavernae* is phylogenetically closely related to *Ovicillium* as pointed by the LSU gene analysis. *Ovicillium* was introduced by Zare and Gams [18] and the genus needed further studies regarding its affinity with *Bionectriaceae.* However, the phylogenetic data obtained in this work are in accordance with the work of Voglmayr and Jaklitsch [27], who verified its phylogenetic position within this family. Although phylogenetically close, *Circumfusicillium* can be easily distinguished from *Ovicillium* [18] by the formation of hyphal ropes and coils (not reported for *Ovicillium*), by the simple phialidic conidiophores (frequently branched with verticillate phialides in *Ovicillium*), by the often-visualized conidia tightly packed by surrounding hyphal segments (not reported for *Ovicillium*) and by the production of long rope-like microsclerotia (not reported for *Ovicillium*). The formation of hyphal ropes and coils, as well as the formation of microsclerotia, have been previously described in some *Bionectriaceae* species parasiting nematodes (e.g., *Ijuhya vitellina* [41]). However, the *C. cavernae* microsclerotia cells *textura globose* are also a distinctive trait (e.g., *textura angularis* in *Ijuhya vitellina* [41]).

GenBank numbers: MUM 20.31 ITS: MT012540; LSU: MT012542. MUM 20.32 ITS: MT012541; LSU: MT012543.

## 4. Discussion

In the present study, the taxonomic status of two unknown *Bionectriaceae* isolates retrieved from a dolomitic limestone wall of the Coimbra’s Roman hypogean cryptoporticus was resolved. The integrative analysis applied allowed the description of a novel genus and species, *Circumfusicillium cavernae* gen. et sp. nov., in *Bionectriaceae*.

The study of cultural heritage materials and the microbial communities responsible for their biodeterioration has seen a high increase in the application of omics methodologies during the last years. Nonetheless, the application of High-Throughput Sequencing (HTS) methods has also highlighted that these relics are inhabited by various unknown microorganisms [42]. Moreover, it is also known that when considering stone structures and monuments, both cultivation and HTS methodologies should be applied in conjunction to achieve complete fungal profiling [21,43]. The cultivation of fungi is thus particularly important, as it also allows the description of previously unknown taxa that can be further studied, taking into account the specific biodeterioration context where they were found. Although their hypothetical contribution to stone biodeterioration processes remains largely unknown, *Bionectriaceae* members have also been found in other European caves and cultural heritage biodeterioration scenarios (e.g., [37,38,39]) and, for example, *Bionectria ochroleuca* was found to be able of in vitro calcite and whewellite mineralization [44].

The phylogenetic analyses conducted with dataset 4 (containing Genbank ITS sequences labeled as “uncultured” and as *Geosmithia* sp. not formally described) also allowed important *Circumfusicillium* ecological characteristics to be inferred, as well as their putative geographical distribution. When considering this data, it is possible to verify that *Circumfusicillium* has been detected throughout the Mediterranean basin. Complementarily, it can also be verified that these microorganisms have been constantly found to be associated to either caves or cultural heritage stone monuments biodeterioration scenarios, highlighting a peculiar and specific ecological characteristic for this genus. Thus far, *Circumfusicillium* has been detected in the Roman cryptoporticus of Coimbra (Portugal); in the Roman Necropolis of Carmona (Spain); in the Andalusian cave Cueva del Tesoro (Spain); in the Dordogne Paleolithic rock art site (France); and in the Gothic building of Santa Maria della Piet (Italy) [37,38,39,40].

While various efforts aiming to explore other gene regions (e.g., the translation elongation 1-α (*tef1*) and the RNA polymerase II subunit 2 (*rpb2*)) in this family have been made, their availability is still limited to only a few genera (e.g., *Geosmithia* and *Clonostachys*). In fact, with the exception of the LSU region, few additional sequence data are available (including from the ITS rDNA region), further hampering proper taxonomic identifications in this family [27,28]. The phylogenetic analyses pointed out that *Circumfusicillium* is closely related to *Ovicillium*; the new genus; peculiar morphological characteristics allow for their distinction. Nonetheless, asexual morphology has been pointed to as a not-so-perfect distinction character for *Bionectriaceae* classification (e.g., [27]) since many species display acremonium-like or verticillium-like characteristics in this family and across the *Sordariomycetes* [18,19]. Considering the *Circumfusicillium* characteristics, this is also verified in this work. On the other hand, regarding the fungus’s peculiar morphological aspects, it should be noted that the *textura globose* formed by the microsclerotia cells is unusual and might be considered an important distinctive trait. Microsclerotia are survival structures that allow fungal survival under various extreme environments, including drought and harsh environmental conditions (e.g., [45]). The formation of microsclerotia by *C. cavernae* likely improves the species’ survival chances, considering the unusual and extreme environment found in the hypogean Coimbra’s Roman cryptoporticus but also in similar caves and stone oligotrophic environments across the Mediterranean basin.

## 5. Conclusions

The previously unknown fungal isolates here described represent a novel genus and species in *Bionectriaceae* and are part of a complex biofilm colonizing the Coimbra’s Roman Cryptoporticus. Thus, this work provides valuable data, increasing the current knowledge of fungi in the order *Hypocreales*, but also in the fungal diversity thriving on hypogean cultural heritage monuments. The description of fungal taxa from cultural heritage monuments is of increased importance, considering that only by knowing the microbial agents involved in the material biodeterioration adequate safeguarding measures can be considered, discussed and applied.

## Figures and Tables

**Table 1:** List of reference isolates considered in the phylogenetic analyses and their respective GenBank accession numbers. The newly generated sequences are presented in **black and bold**.

Species	Isolate Reference	LSU Accession Number	ITS Accession Number
*Acremonium acutatum*	CBS 682.71	NG056976	MH860300
*Acremonium alternatum*	CBS 407.66	NG056977	MH424672
*Acremonium fusidioides*	CBS 840.68	NG056984	FN706542
*Acremonium hennebertii*	CBS 768.69	NG056987	MH859420
*Acremonium sclerotigenum*	CBS 124.42	NG057139	MH856101
*Acremonium zeylanicum*	CBS 746.73	HQ232154	-
*Bryocentria brongniartii*	M190	EU940125	-
*Bryocentria metzgeriae*	M140	EU940106	-
*Bulbithecium hyalosporum*	CBS 318.91	AF096187	NR_137155
*Bullanockia australis*	CPC 28976	KY173506	-
*Caespitomonium euphorbiae*	CPC 39083	OK663737	OK664698
***Circumfusicillium cavernae***	**MUM 20.31**	**MT012542**	**MT012542**
***Circumfusicillium cavernae***	**MUM 20.32**	**MT012543**	**MT012543**
*Clonostachys epichloe*	CBS 118752	DQ363259	-
*Clonostachys grammicospora*	GJS 85-218	AF193238	-
*Clonostachys ochroleuca*	CCFC 226708	AY283558	-
*Clonostachys pityrodes*	GJS 95	AY489728	-
*Clonostachys setosa*	CBS 834.91	AF210670	AF210670
*Emericellopsis glabra*	AR 3614	GQ505993	HM484860
*Emericellopsis maritima*	AFTOLID 999	FJ176861	-
*Emericellopsis terricola*	CBS 120.40	U57082	MH856058
*Geonectria subalpina*	CBS 143540	MH155487	-
*Geosmithia langdonii*	CCF 4326	-	KF808298
*Geosmithia lavendula*	CBS 582.67	KT155289	MH85905
*Geosmithia microcorthyli*	602a	-	MT955334
*Geosmithia putterillii*	CBS 260.33	KT155185	MH855435
*Gliomastix masseei*	CBS 794.69	HQ232060	MH859431
*Gliomastix roseogrisea*	CBS 279.79	HQ232122	-
*Heleococcum aurantiacum*	CBS 201.35	JX158441	MH855645
*Heleococcum japonense*	CBS 397.67	JX158442	JX158420
*Heleococcum japonicum*	ATCC 18157	U17429	-
*Hydropisphaera peziza*	GJS 92101	AY489730	-
*Hydropisphaera suffulta*	CLLMAR 13023	KU237207	-
*Ijuhya chilensis*	CBS 102803	KY607553	KY607538
*Ijuhya fournieri*	CLLG10113	KP899118	-
*Ijuhya peristomialis*	CBS 569.76	KY607559	KY607544
*Kallichroma glabrum*	JK5123	AF193233	-
*Kallichroma tethys*	JK5181	AF193234	-
*Lasionectriella rubioi*	CBS 140157	KU593581	-
*Leucosphaerina arxii*	CBS 737.84	NG057892	NR_145040
*Mycoarachis inversa*	ATCC 22107	NG059437	HM484861
*Nectriopsis violacea*	MUCL 40056	AF193242	-
*Nigrosabulum globosum*	ATCC 22102	AF096195	NR_160124
*Ochronectria calami*	ATCC46692	AF193243	-
*Ochronectria thailandica*	MFLUCC 15-0140	KU564069	KU564071
*Ovicillium attenuatum*	CBS 158.96	KU382232	KU382186
*Ovicillium napiforme*	CBS 426.95	KU382233	KU382192
*Ovicillium oosporum*	CBS 110152	KU382234	KU382194
*Ovicillium subglobosum*	CBS 101963	KU382235	NR_154335
*Paracylindrocarpon aloicola*	CPC 27362	KX228328	KX228277
*Protocreopsis korfii*	CLLM 14077	KT852955	-
*Protocreopsis pertusa*	CTR 72184	GQ506002	-
*Roumegueriella rufula*	GJS 91164	EF469082	-
*Selinia pulchra*	AR 2750	AF193246	-
*Stephanonectria keithii*	GJS 92133	AY489727	-
*Stilbocrea colubrensis*	CLLM 16003	MN497409	NR_173884
*Stilbocrea gracilipes*	CLLM 16015	-	MN497407
*Stilbocrea macrostoma*	GJS 02125	GQ506004	-
*Stilbocrea macrostoma*	GJS 7326	AY489725	-
*Stilbocrea macrostoma*	WU 32032	MH562718	-
*Stilbocrea walteri*	CBS 144627	MH562717	NR_160063
*Stromatonectria caraganae*	CBS 127387	HQ112287	HQ112287
*Synnemellisia aurantia*	COAD 2070	KX866396	NR_154444
*Verrucostoma freycinetiae*	MAFF 240100	GQ506013	NR_137761
*Verrucostoma martiniciensis*	PAM 2015	KP192672	-
*Xanthonectria pseudopeziza*	CLL16005	KU946964	-

## Data Availability

Generated DNA sequences are available in GenBank (accession numbers MT012540-MT012541 and MT012542-MT012543). All the phylogenetic alignments and trees obtained are available in figshare: 10.6084/m9.figshare.20279658.
